# Value Conflicts in Designing for Safety: Distinguishing Applications of Safe-by-Design and the Inherent Safety Principles

**DOI:** 10.3390/ijerph18041963

**Published:** 2021-02-18

**Authors:** Britte Bouchaut, Lotte Asveld, Ulf Hanefeld, Alexander Vlierboom

**Affiliations:** Department of Biotechnology, Delft University of Technology, Van der Maasweg 9, 2629 HZ Delft, The Netherlands; l.asveld@tudelft.nl (L.A.); u.hanefeld@tudelft.nl (U.H.); A.C.E.Vlierboom@student.tudelft.nl (A.V.)

**Keywords:** safe-by-design, inherent safety principles, lock-ins, values, biochemistry, biotechnology

## Abstract

Although both the Inherent Safety Principles (ISPs) and the Safe-by-Design (SbD) approach revolve around the central value of safety, they have a slightly different focus in terms of developing add-on features or considering initial design choices. This paper examines the differences between these approaches and analyses which approach is more suitable for a specific type of research—fundamental or applied. By applying the ISPs and SbD to a case study focusing on miniaturized processes using Hydrogen Cyanide, we find that both approaches encounter internal value-conflicts and suffer from external barriers, or lock-ins, which hinder implementation of safety measures. By applying the Technology Readiness Levels (TRLs), we gain insight in the matureness of a technology (thereby distinguishing fundamental and applied research) and the extent of lock-ins being present. We conclude that the ISPs are better able to deal with lock-ins, which are more common in applied research stages, as this approach provides guidelines for add-on safety measures. Fundamental research is not subject to lock-ins yet, and therefore SbD would be a more suitable approach. Lastly, application of either approach should not be associated with a specific field of interest, but instead with associated known or uncertain risks.

## 1. Introduction

One of the most acknowledged values in the fields of chemical engineering, biochemistry and biotechnology is safety. To ensure (bio)chemical processes to be acceptably safe for society, animals and the environment, multiple approaches have been developed over the last decades. Examples of such are the 12 principles of Green Chemistry [1,2], Safety Management Systems [3], Inherent Safety [4] and the Inherent Safety Principles (ISPs) [5]. In the field of chemical engineering, in particular, the ISPs are widely known [6] and aim at eliminating or minimizing the risks of hazardous chemicals or syntheses by using conditions or chemicals with less dangerous properties. The Safe-by-Design (SbD) approach, which is derived from the notion of inherent safety, has been gaining foot in the field of nanotechnology [7,8], biotechnology and synthetic biology [9,10] over the last decade. Although both approaches revolve around measures for safety, the derived measures differ to some extent. That is, the ISPs provide guidelines for risk-reducing measures or the development of add-on safety features [5,11], while SbD questions the initial use of certain chemicals or carriers during the early stages of development more strongly [12]. However, although there is a difference between the derived measures, both approaches suffer from internal value-conflicts (e.g., safety vs. performance or sustainability) during implementation [13,14]. However, in terms of lock-ins—external barriers such as company culture or established infrastructure that hinder implementation or adoption of an alternative process or technology—mostly SbD is affected. As the ISPs provide add-on safety measures [5,11], they would be, to a certain extent, able to take lock-ins into account. The SbD approach, however, may call for more drastic changes in terms of design choices (e.g., choice of raw materials) and can therefore experience more hindrance of external barriers (e.g., would call for a change in process set-up and/or infrastructure). In addition, although the approaches differ in optional measures for safety, there also seems to be some overlap. For example, it could be argued that the SbD strategy of developing kill switches [12] also fits within one of the ISPs as its goal is to reduce any possible negative consequences might anything unforeseen happen. The other way around, the ISP of substitution—the replacement of hazardous chemicals with less hazardous ones [4,5]—could also be classified as a SbD strategy.

Although the ISPs are considered an already established approach for risk reduction and SbD is considered a relatively new approach, the distinction between these approaches seem to be somewhat blurry. Therefore, this paper aims to define the differences between these approaches and to shed light on which approach would be better applicable to a specific type of research: either applied or fundamental research. Although internal conflicts occur in both types of research, external conflicts such as lock-ins are more heavily present in applied research stages. Therefore, either approach might be better able to deal with a specific type of conflict.

In order to analyze these differences and the applicability of both approaches, we have chosen a case study from the field of biochemistry that focuses on the miniaturization of processes (i.e., the use of micro-reactors) using Hydrogen Cyanide (HCN) [15,16,17]. HCN, a commonly used C1-building-block within industry, is an extremely toxic compound for humans and animals with possibly lethal consequences when exposure occurs in low concentrations [18]. However, the compound also comes with great benefits in terms of its low number of by-products, its broad applicability for syntheses (due to it only having one carbon atom), and its relatively easy and cheap production. By applying the concept of miniaturization, this leads to an increase in (industrial) safety as the smaller reactors assure that less toxic cyanide would be present at any given time. Therefore, the idea of minimization, one of the ISPs, lowers the hazard (i.e., exposure to a lethal dose of cyanide) and therefore the associated risk. However, the notion of SbD would already question the initial usage of such an extremely toxic compound and would encourage using or searching for alternatives that would be less toxic [10]. Considering that HCN is widely used in industry and has been since its discovery by the end of the 18th century, currently, there are hardly any alternatives (with the same properties and similar benefits), and incentives for researching alternatives seem to be lacking. Especially the latter sheds light on the applicability of the ISPs and SbD to already established syntheses and processes and raises the question of which approach would be more suitable for different research stages.

This paper is structured as follows. First, we introduce miniaturization processes using HCN and provide an overview of the concepts of inherent safety, the ISPs and SbD. Second, by applying either approach to the case study, this sheds light on their applicability for a specific research stage. We identified some internal value-conflicts in terms of *safety*, *sustainability*, and *efficiency*, and external conflicts or *lock*-*ins*, such as (company) culture and already established safety measures. These results indicate that multiple values should be taken into account when designing for safety and that either approach differs in their applicability for a specific research stage. By applying Technology Readiness Levels (TRLs) specifically defined for the chemical industry [19], we can identify the technology’s development stage and whether a product or process may already suffer from certain barriers or lock-ins that might lead to value conflicts in choosing measures for safety. We argue that SbD would be more suitable for early-stage development or fundamental research (TRLs 1–5). As applied research (TRLs 5–9) may already suffer from lock-ins, this complicates application of the SbD approach, and the ISPs would be more appropriate here. Last, we conclude that neither of the approaches should be associated with a specific domain, but instead with the emergence of known or uncertain risks.

## 2. Methods

This study comprises three components: (1) A literature study focusing on inherent safety, the ISPs and SbD, (2) semi-structured interviews that helped to clarify the specific context of the case study regarding miniaturized processes using HCN, and (3) analysis of the suitability of the ISPs and SbD by applying either approach to the case study. This means that this study comprises an empirically informed conceptual analysis, in which the conducted interviews mostly provided information concerning the miniaturized processes, and what it would entail to implement this type of technology in industry. The analysis part of this study is mostly based on existing literature from which we defined the relevant concepts for this study, but complemented with information derived from the interviewees.

The reason we chose miniaturized processes using HCN as a case study is the availability of comprehensive knowledge and of many safety procedures that make it possible to work with this compound safely. However, coming from a SbD-perspective, we might be questioning whether we should be working with such a hazardous substance at all considering its lethal properties. This case study allows us to research what effects applying the SbD approach would have, what its bottlenecks would be and what the differences are between applying the ISPs and SbD in practice.

In terms of empirical input, interviews (N_tot_ = 7) were conducted from October to December 2019 with a range of relevant stakeholders that gave more information about (technical) details of the miniaturization of HCN processes itself, (national) regulation in terms of safety measures from a governance and knowledge institution’s perspective, the current usage of HCN within industry and whether and which values are at stake for different stakeholders. Interviewees from academia are employed as a Principal Investigator (PI) (N = 2), PhD researcher (N = 1) and a Safety Officer (N = 1). Furthermore, two interviews were conducted with representatives of a global industrial (bio)chemical concern (BCM1; BCM2), and one interview with a risk assessor employed by the Dutch National Institute for Public Health and the Environment (In Dutch: Rijksinstituut voor Volksgezondheid en Milieu (RIVM)).

The interviews followed a semi-structured approach that left enough room for interviewees to go into detail when the researchers felt this was necessary for clarification or context. The interviewees were selected based on their experience in the domain of (bio)chemistry and field of profession. In addition, all interviewees hold senior positions, except for the PhD researcher. At the start of each interview, we asked the interviewee to sign a form of consent to approve recording the interview. After the interview, a transcript was sent to the interviewee for any remarks or corrections. Upon receiving the interviewee’s approval, the transcript was anonymized, coded and analyzed. All data (i.e., form of consent, interview protocol, interview transcripts) can be requested from the corresponding author (see Data Availability Statement). 

## 3. Cyanide Research

C-1 chemistry entails the field of research that uses one-carbon reagents. Examples of such are methane (CH_4_), carbon monoxide (CO), methanol (CH_3_OH) and hydrogen cyanide (HCN). In particular the latter is considered the cheapest and most versatile building block within this specific domain [20]. However, although this compound comes with great benefits such as its low costs and relatively easy production coupled with its high efficiency for syntheses with a low number of by-products, it also poses a health threat due to its toxic properties. That is, compared to other toxic gasses (e.g., CO), its lethal concentration is extremely low, i.e. HCN: 110 mg/m^3^ [21] compared to CO: 2.000 mg/m^3^ [22]—10 min exposure meaning that the chance of a fatality is very high. Therefore, many safety measures and procedures have been developed such as the usage of closed reactors or specific safety protocols and equipment to handle HCN safely. More recently, increasing interest is given not only to novel techniques for safety but also to positively impact the energy efficiency and environmental aspects. One of such techniques is *miniaturization*; a type of process intensification that leads to substantially smaller and more efficient chemical processes and synthesis pathways [23].

### Miniaturization

**Figure 1:**
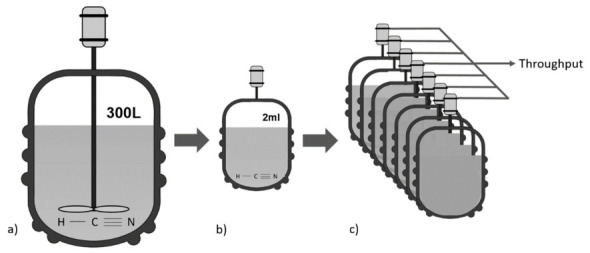
Simplified, schematic representation of the concept of miniaturization. (**a**) Illustration of a batch reactor (300 L), (**b**) a miniaturized reactor (2 mL), and (**c**) outscaling—the coupling of multiple micro-reactors in parallel for higher throughput.

As the name ‘miniaturization’ already implies, micro-reactors are used to intensify processes and to reduce the scale of equipment (Figure 1a,b). In these types of processes, low(er) volumes (e.g., 500 µL–2 mL) are used that make it possible to, for example, enable reactions under higher temperature or using higher concentrations, as well as better process control and heat management [23,24]. Furthermore, as micro-reactors require fewer materials, equipment and installation costs would be lower, fewer demands in laboratory infrastructure would be necessary, better process performance could be achieved, and the process by itself would be inherently safer [25]. In particular, the latter is advantageous when working with highly toxic compounds.

As micro-reactors make the overall process more controllable [15], these are especially interesting for industry, in particular when *outscaling* is applied. This means that several micro-reactors are coupled in parallel (Figure 1c) in order to achieve higher throughput or a larger production volume. Due to the improved controllability per micro-reactor, this also leads to a higher level of safety [15]. In addition, miniaturized processes are also generally regarded as highly efficient because batch processes (a finished lot or quantity after one production cycle) can be converted into miniaturized continuous systems. However, converting batch processes into continuous processes also calls for finding an effective method of immobilization for the used enzyme, which can be problematic.

## 4. Designing for Safety

Miniaturized processes can be regarded as a safer alternative to already established syntheses and processes as they provide a lower volume of HCN to be present at any given time. However, as HCN is still being used, the risk of incurring a lethal dose is still present. In that sense, a ‘true’ inherently safe design would not make use of any hazardous substances in the first place, which is exactly the idea behind the SbD approach.

Within the chemical industry, safety measures are generally applied to already existing techniques and are mostly focused on technicalities. In general, these measures are classified as (1) engineered safety, (2) procedural safety, and (3) inherent safety [11]. Engineered safety involves add-on safety features that do not perform any fundamental operation within the process itself, but only become active when an issue within the process occurs. Procedural safety entails measures for safety, such as safety protocols, that reduces risks for safe work practices. Inherent Safety comprises using the properties of a material or process to eliminate or reduce the hazard (i.e., the potential for harm) itself [4]. Given the definition of risk (i.e., risk = hazard * probability), this means that the risk is also lowered although the probability that anything might happen would remain the same. This reduction or elimination of hazards is also exactly what makes inherent safety different from engineered or procedural safety; it seeks to minimize the hazard at the source instead of accepting the hazard and taking add-on safety measures [11].

### 4.1. Inherent Safety

In order to approach inherent safety, the Inherent Safety Principles (ISPs) have been developed, which, mostly with a technical approach, function as guidelines for safe product and process design [4,5]. The four general principles are (1) *Minimization*: Using smaller quantities of hazardous substances, (2) *substitution*: Replacing hazardous chemicals with less hazardous ones, (3) *moderation*: Using less extreme reaction conditions, a less hazardous form of a material or use facilities that minimize the impact of a hazardous material, and (4) *simplification*: Designing facilities in such a way that any unnecessary complexity is eliminated and makes operating errors less likely to occur. Although all four principles have the goal of making products or processes safer, these cannot be applied simultaneously [5,13,26,27]. As we will elaborate in Section 5.1, applying miniaturized processes using HCN (the ISP of minimization) can be deleterious for other ISPs, thereby leading to internal conflicts.

### 4.2. Safe-by-Design

Safe-by-Design (SbD) is an approach for (experimental) process design focusing on procedural and technical risk management and is currently gaining foot in the fields of nanotechnology and biotechnology [7,10,26]. Although compared to the ISPs, SbD has a more socio-technical approach as it encourages active stakeholder engagement and communication about design choices and implementing measures for safety, associations with both approaches seem to overlap [9] as they both refer to the idea of designing specifically for safety by integrating knowledge about the adverse effects of materials in the technology’s design process [13]. However, when applying the ISPs, it is assumed that sufficient knowledge is available about the adverse consequences or risks of using such chemicals or production routes—as illustrated in the previous section. As SbD already questions the initial use of hazardous chemicals and the design principles are solely focused on the value of safety, SbD tends to focus more on issues related to uncertain risks [28]. For example, technologies that are still under development can be prone to uncertain risks as they have not reached a certain level of matureness to oversee all possible consequences. When knowledge about possible consequences turns out to be insufficient, the SbD approach can enable an iterative process in which many stakeholders are involved. That way, a range of different issues can be addressed, reflected on and incorporated in design choices, coming to a collective design with safety in mind [9]. Therefore, in contrast to the ISPs that mostly have a technical focus, SbD can also incorporate socio-technical implications. However, this also means that although SbD can initially put more weight on the value of safety, later, other values such as sustainability might become relevant too as we will elaborate in Section 5.2.

## 5. Comparative Analysis

This paper aims to define the differences between the ISPs and SbD and which approach would be more suited for either fundamental or applied research. First of all, the conducted interviews helped to clarify the specific context in terms of our case study. Following that, by applying either approach to our case study and based on literature, we found that both the ISPs and SbD suffer from internal conflicts and external barriers, or lock-ins. However, in terms of the latter, we found that SbD finds more hindrance from these lock-ins and the ISPs would be more able to deal with these as they provide ‘add-on’ measures for safety, in comparison to SbD. For the sake of clarity, this study entails an empirically informed conceptual analysis, meaning that the presented results in this section are partly derived from the conducted interviews (context) and supported by literature (concepts) (All data is available upon request, see Section 2).

### 5.1. Internal Conflicts within the ISPs

As already touched upon in Section 4.1, not all ISPs can be applied simultaneously as this would cause internal conflicts. In the following sections, we provide a deeper analysis of occurring value-conflicts in line with [13] and [5], who have described these extensively. Using our case study, analysis of these conflicts illustrates what trade-offs would have to be made to achieve an inherently safer design from a technical perspective and whether this would be feasible. Besides, the latter also sheds light on the applicability of the ISPs in terms of such internal conflicts.

#### 5.1.1. Inherent Safety vs. Performance

Inherently safer chemicals or synthesis pathways might not always perform to the same extent as less safe alternatives. However, whether something can be deemed more efficient is dependent on what the comparison is made with, which also applies to miniaturization processes using HCN. For example, when such processes are compared with batch processes, miniaturization can indeed contribute to a more efficient (and safer) process. Batch processes are most commonly used for applications that have to be made under sterile conditions such as raw materials for food supplements. Therefore, such processes are conducted in a closed reactor vessel in which no substances are added or discarded during synthesis except for oxygen for pH adjustment. However, due to the mixing/stirring of substances in the vessel, heat is being released, which can affect process efficiency. If we would move from batch reactors to miniaturized, continuous flow processes, the efficiency would indeed increase as no energy would be required for stirring anymore and the temperature within the vessel would remain constant, meaning that no energy would be required for cooling.

Although miniaturized processes could help us improve safety, interviewees pointed out that a trade-off between safety and other relevant values would have to be made when transitioning to miniaturized processes. For example, industry already using continuous processes would take little or no benefit from miniaturization in terms of process efficiency. In addition, according to interviewees from a global (bio)chemical company, production routes and syntheses performed in industry are already deemed safe. As these firms have to comply with regulation, provide training for their staff and apply preventive safety measures to ensure a responsible and safe work environment, a question to them would be how much could be gained in safety when miniaturized processes would be implemented, and at what cost? In addition, according to the interviewees, if mini-reactors would be used, it would become more difficult to monitor the quality of raw materials with possible negative effects for the end-product’s quality.

#### 5.1.2. Inherent Safety vs. the Environment

Miniaturized processes can contribute to more environmentally friendly processes as they are more efficient and therefore lower amounts of toxic chemicals are used. However, as the CN-groups from HCN would still be inherently toxic, alternatives should be sought in order to contribute to a safer environment. For HCN, alternative forms can indeed be found that would expose a lower risk, for example, forms where the CN-groups would be retained to salts such as potassium hexacyanoferrate (III) (K_3_Fe(CN)_6_) or potassium hexacyanoferrate (II) (K_4_Fe(CN)_6_) [29]. As the CN-groups form a strong bond with the iron in these salts, in theory, these would even be safe enough to be consumed by humans. However, as was pointed out by interviewees (PI1, PI2), in terms of the environment, to break the strong bond between the iron and the CN-groups, more extreme reaction conditions are required such as a higher temperature (and thus more energy) and a higher pH, which can lead to the formation of more residual products, which would not be favorable from a sustainability perspective. Besides, a higher temperature might lead to certain enzymes no longer functioning when an enantiomer (optical isomer—right- or left-handed) is targeted. Because of this, the suggested alternatives K_3_Fe(CN)_6_ and K_4_Fe(CN)_6_ would be limited to only a number of syntheses or could only be used when a racemic mixture (equal parts of optical isomers) is targeted and enzymes are not required.

#### 5.1.3. Inherent Safety vs. the Inherent Safety Principles

Given the limited range of alternatives to HCN, and that the ones available may be at the expense of other relevant values (i.e., energy efficiency, sustainability), it is clear that application of the ISPs can lead to internal value-conflicts. As already touched upon in Section 4.1 and Section 5.1.2, substitution of HCN with, for example, CN-salts would require more energy, would call for more extreme reaction conditions (i.e., higher temperature) and could result in more residual products. The same conflict occurs within one of the ISPs itself; moderation. Although we would be using a less hazardous material, this would not result in using less extreme reaction conditions. Of course, we could also exclude using CN-groups and search for other C-1 chemicals such as CO or CH_4_. However, as these compounds also have toxic properties and are harmful to the environment, these would still be deleterious in terms of the other ISPs.

#### 5.1.4. Hazard vs. Hazard

Other, alternative compounds could also just induce different hazards. For example, we could also be using sodium cyanide (NaCN), which is far less hazardous than HCN and would create a safer environment for laboratory personnel to handle this compound. However, in an acidic environment, NaCN could easily form the gaseous HCN and still pose the same risk. Therefore, researchers need to assure that all work is conducted in a basic environment (pH > 11), which would require extra control measures, thereby also creating the probability for potential failure.

### 5.2. Internal Conflicts within Safe-by-Design

Technical designs often have to fulfil more requirements than, in this case, solely safety. In terms of SbD, as this approach places more weight on the value of safety, this can turn out to be detrimental for other values. For example, using CN-salts such as K_3_Fe(CN)_6_ or K_4_Fe(CN)_6_ [29] as described in Section 5.1.2. These compounds might be safer in terms of usage, they turn out to be deleterious in terms of sustainability. Such internal value conflicts would call for a trade-off [30]. In that sense, we can assign two distinctions of applying SbD: Product-applied and process-applied [9]. Product-applied SbD entails safety measures specifically applied upstream, aimed at the technical components or the product itself. Process-applied SbD entails measures applied downstream, aimed at design decisions regarding scaling-up and further implementation. In terms of value trade-offs, transferring from product- to process-applied SbD might call for a different balance (i.e., safety vs. sustainability). However, in terms of creating inherent safety, safety would still be the core value at stake. If certain design requirements would call for a value trade-off, this would also imply that we would possibly have to ‘give in’ on safety. Although designers often have to accept such a trade-off for certain reasons, they could also look for new or alternative technical options minimizing the trade-offs that would have to be made [30].

### 5.3. Lock-ins

Application of both approaches to our case study of miniaturized processes already illustrated some internal conflicts and value trade-offs, which are mostly technically focused. However, devoting research to alternative, inherently safe raw materials (SbD approach) or implementation of miniaturized processes (ISP) also encounters other barriers than solely technical ones. Based on conducted interviews with representatives from industry, these barriers were identified as company culture, infrastructure, regulation and IPR, to which we refer as *lock-ins*.

From a company’s perspective, devoting research to and eventually implementing alternative production methods or radically different synthesis pathways requires investments. However, when existing methods or pathways are already considered satisfactory in terms of their efficiency, costs, safety and the end-product’s quality, and it is yet not clear what an alternative could add to one of these factors, incentives could be lacking [14]. In addition, although the industrial sector has been paying attention to creating inherently safe(r) processes over the past years, interviewees from industry pointed out that some companies may have outdated plants and installations. They mention that investments in infrastructure are often made for 20–40 years, and measures for safety are often add-on measures to already existing processes and conditions. If one would like to take a very different path, for example by implementing miniaturized processes and outscaling, this would not always be possible for existing plants. These, or other even more radically different processes could be best implemented when building a new production site, but this would require a consensus (BCM1). As corporate cultures are not always set to make fast decisions on such rigorous changes, accepting and implementing these changes often takes more organizational time (BCM1; BCM2). Along the lines of these barriers, [26] argues that inherent safety is a radical departure from the traditional approach of looking at additional safety features first, as recommended by conventional safety codes and standards. Therefore, time would be needed to encourage people to change their thinking and practice to create inherent safety.

Creating inherent safety would take more than holding on to existing safety codes and standards as people’s behavior and actions can also influence safety. For example, the more experienced people get, the more they learn and can become (more) aware of any induced risks, leading to behaving in a certain way and adhering to safety protocols. However, more experience could also lead to habituation where people spend less attention to, or disobeying protocols. “*A researcher working with HCN for the first time will be more attentive than someone who has done this already a 100 times*”(PI2). From a SbD perspective, experience can be of great importance for creating an inherently safer environment. As SbD encourages stakeholder involvement [8], more discussion and engagement between relevant parties is invited, and more experience is brought in to anticipate potential risks [31]. As a broad range of stakeholders can share their vision and perspectives, measures could be designed collectively that would anticipate a wide range of potential risks. In terms of open communication for the sake of safety, mostly the domains of healthcare and the aviation industry are described in literature [32,33], in particular creating awareness and developing anticipatory measures such as ‘learning from each other’. As parties can share data and information about, for example ‘almost incidents’, better, faster and anticipatory solutions can be developed [34]. However, although the chemical industry would like to transition to a more ‘open’ culture, many seem to struggle to enable such (ibid.). Interviewees (BCM1; BCM2) indicated that companies tend to be reluctant in being open and transparent—*“they do not necessarily feel the need to share information with others“*. The reason they give for this lack of transparency is that they possess all the necessary expertise and experience to be able to deal with safety measures responsibly. In addition, related to patent due, any information released could lead to ownership issues, jeopardizing patent filing.

### 5.4. Differentiating the ISPs and SbD

Working towards inherently safer products and processes turns out to be not so straight-forward and depends on many factors such as people’s way of thinking and acting, work culture and certain lock-ins such as infrastructure and IPR. When comparing application of the ISPs to the SbD approach, the ISPs offer more technically oriented risk-reducing measures. Therefore, the ISPs would be a better fit to deal with lock-ins as they provide guidelines that already take into account certain initial product- and process design choices (i.e., choice of chemicals, synthesis pathway, plant design). SbD calls for a different attitude to critically (re)think initial design choices (i.e., searching for alternatives to highly toxic chemicals). Therefore, we argue that SbD is more about inherent safe design while the ISPs focus more on safe process design.

## 6. Assigning Types of Research

Although the chemical industry is often more associated with applied research and knowledge institutions such as academia with fundamental research, we must not simply base the suitability of the ISPs and SbD on this association. Instead, we should look at a technology’s development stage and the rise of known or uncertain risks to distinguish the approaches’ applicability.

### 6.1. Technology Readiness Levels

The Technology Readiness Levels (TRLs) could offer a systematic structure that supports assessment of the maturity of technologies for the chemical industry [19]. Within this study, we build upon the TRLs specifically defined for the chemical industry (Table 1) by [35].

Building upon the defined TRLs and descriptions provided in Table 1, it is important we first make a distinction between *fundamental* and *applied research*. For the levels 1–5, we feel that fundamental research would be more fitting as the technology is still in its early developmental stage within laboratory settings, thereby giving rise to more uncertain risks. In addition, we do acknowledge a difference for levels 1–3, which are mostly technically focused in terms of design choices, and levels 4–5, which also entail preparations for developing process design and scaling-up. The levels 5–9 consist of more advanced testing of process design, pilot trials and the operation of full-scale plants, which we associate more with known risks and applied research. Therefore (and recalling Section 5.4), we associate the TRLs 1–5 more with inherent safe design as it entails early (experimental) design choices (SbD approach) that would make the product or process already inherently ‘safe’, and the TRLs 5–9 more with safe process design (ISPs) as it involves add-on measures for safety.

**Figure 2:**
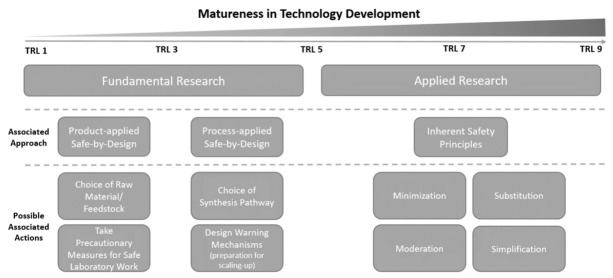
Defined Technology Readiness Levels with the associated type of research, approach (Safe-by-Design (SbD) or the Inherent Safety Principles (ISPs)) and possible safety measures to take.

In terms of assigning a fitting approach to the early research stages, the SbD approach can also make a distinction between the early developmental stages (TRLs 1–3) and the early process design (TRLs 4–5). In that sense and recalling Section 5.2, we can assign product-applied, and process-applied SbD strategies [9] such as the choice of raw material (e.g., chemicals) or develop built-in warning mechanisms might anything unforeseen develop. That would imply that for all TRLs, the value of safety is most prominent, but a balance could be found with other values that might become relevant when transitioning to later stages (TRLs 4–5), such as the values of sustainability or efficiency. For research that would be classified TRLs 5–9, certain design choices have already been established in e.g., infrastructure (existing chemical plants). Therefore, implementing measures for safety should be able to take these into account, and application of the ISPs would be more suitable here. For clarity, we constructed Figure 2, which illustrates the distinction between the types of research based on the TRLs, and their associated approaches and possible measures for safety.

### 6.2. Applicability to Domains

Although the field of chemical engineering is generally more associated with applied research and ‘newer’ fields such as biotechnology and nanotechnology more with fundamental research, application of either the ISPs or the SbD approach should not be decided upon the specific domain but should be considered on the stage of research and what types of risk arise.

The domain of chemical engineering constitutes a more traditional field with decades of knowledge and experience. Therefore, this field, and in particular process design, is often more associated with applied research and known risks, which makes it very suitable for application of the ISPs. For the fields of biotechnology and synthetic biology, the SbD approach has been gaining foot [10,36,37]. As these fields are ‘newer’ compared to the chemical domain, this can give rise to uncertain risks. For example, unexpected operating conditions during bio-energy production causing the release of hazardous substances [38], or the possibly accidental release and spread of synthetic cells and carriers [39,40,41,42]. However, uncertain risks are not solely limited to ‘new’ domains of engineering, but can also still arise in the domain of chemical engineering, e.g., pesticides or PFOA [43]. Therefore, neither of the approaches should be associated with a specific domain as known and uncertain risks also do not limit themselves to a specific field of interest.

## 7. Conclusions

This paper aimed to define the differences between the ISPs and SbD and to shed light on which approach would be better applicable to what type of research: Either applied or fundamental research. For both approaches, we identified internal conflicts and external lock-ins that called for some value trade-offs. However, especially SbD appeared to be less able to cope with external barriers in comparison to the ISPs as they provide guidelines for add-on safety measures. In contrast, as SbD assigns more weight to the value of safety in early design choices, this can lead to more radical measures for safety. Therefore, we argue that SbD is more about inherent safe design while the ISPs focus more on safe process design.

Our case study on miniaturized processes using HCN illustrated that a trade-off within the ISPs can only be made when risks (and benefits) are known. As known risks are more associated with applied research (TRLs 5–9), we argued that the ISPs would be more suitable for this type of research as they take into account certain lock-ins and provide guidelines for safety measures from thereon. In case of uncertain risks, making a trade-off between the ISPs would be impossible. As SbD encourages stakeholder involvement and calls for a different attitude to critically (re)think initial design choices, this approach would be more suitable for early-stage, or fundamental research (TRLs 1–5). Although taking appropriate measures to anticipate uncertain risks is challenging, it could give the opportunity to already find the safest possible pathway at the beginning of a technology’s development. As it does not suffer from lock-ins (yet), this could help to create incentive for devoting research to alternatives.

### Concluding Remarks

This study entails an empirically informed conceptual analysis, meaning that the conducted interviews mostly functioned to gain understanding of the relevant context (i.e., miniaturized processes, safety measures and possible barriers for implementation from an industry perspective). The interviews were mostly carried out with people employed in the Netherlands (e.g., Dutch research institute) although interviewees did have different nationalities and working experience outside the Netherlands. The interviewees from a global biochemical firm have senior international experience and are not stationed in the Netherlands. Therefore, the knowledge derived from these interviews is partly based on Dutch regulation (i.e., Safety Officer complying with Dutch legislation) but not limited to this. In addition, all interviewees were from within the EU, so the overarching set of rules is identical. Furthermore, technicalities or process design related to miniaturized processes or syntheses using HCN are not limited to a specific country or region and are therefore representative of safety and design issues, even beyond the European context.

Safety is and will remain a contentious issue within the chemical and biotechnical domain, and does not only encompass technicalities or safety measures in terms of process and/or plant design. Although procedural safety aims to capture human behavior (and failure) to a large extent, human mistakes cannot be fully omitted. In terms of future research, Artificial Intelligence (AI) and machine learning could be implemented for processes where human behavior is a concern. However, such automated processes could also give rise to a new dimension with regard to engineered safety, in case such systems fail and would be in need of human interference.

## Figures and Tables

**Table 1:** Definition of Technology Readiness Levels (TRLs) for the Chemical industry. Adapted from [35].

TRL	1	2	3	4	5	6	7	8	9
Title	Idea	Concept formulated	Proof of Concept	Preliminary Process Development	Detailed Process Development	Pilot Trials	Final Engineering	Commissioning	Production
Description	Opportunities identified, basic research translated into possible applications (e.g., by brainstorming, literature study).	Technology concept and/or application formulated, patent research conducted.	Applied laboratory research started, functional principle/reaction (mechanism) proven, predicted reaction observed (qualitatively).	Concept validated in laboratory environment, scale-up preparation started, conceptual process design (e.g., based on simulation with simple models).	Shortcut process models found, simple property data analysed, detailed simulation of process and pilot plant using bench scale information.	Pilot plant constructed and operated with low rate production, products tested in application.	Parameter and performance of pilot plant optimized, (optional) demo plant constructed and operating, equipment specification including components that are type conferrable to full-scale production.	Products and processes integrated in organizational structure (hardware and software), full-scale plant constructed, start-up initiated.	Full-scale plant audited (site acceptance test), turn-key plant, production operated over the full range of expected conditions in industrial scale and environment, performance guarantee enforceable.
Workplace	Sheets of paper (physical or digital), whiteboard or similar.	Sheets of paper (physical or digital), whiteboard or similar.	Laboratory.	Laboratory/ Miniplant.	Laboratory/ miniplant.	Pilot plant, technical centre.	Pilot plant, technical centre, (optional) demo plant (potentially incorporated in production site).	Production site.	Production site.

## Data Availability

All data can be accessed via https://doi.org/10.17026/dans-x9n-prcm (accessed on 14 February 2021).

## References

[1] Anastas P., Warner T. (1998). Green Chemistry: Theory and Practice.

[2] Anastas P., Eghbali N. (2010). Green chemistry: Principles and practice. Chem. Soc. Rev..

[3] Reniers G.L.L., Ale B.J.M., Dullaert W., Soudan K. (2009). Designing continuous safety improvement within chemical industrial areas. Saf. Sci..

[4] Kletz T. (1996). Inherently Safer Design: The Growth of an Idea. Process Saf. Prog..

[5] Khan F.I., Amyotte P.R. (2003). How to Make Inherent Safety Practice a Reality. Can. J. Chem. Eng..

[6] Kletz T.A. (2003). Inherently safer design its scope and future. Process Saf. Environ. Prot..

[7] Kelty C.M. (2009). Beyond Implications and Applications: The Story of “Safety by Design”. Nanoethics.

[8] van de Poel I., Robaey Z. (2017). Safe-by-Design: From Safety to Responsibility. Nanoethics.

[9] Bouchaut B., Asveld L. (2020). Safe-by-Design: Stakeholders ’ Perceptions and Expectations of How to Deal with Uncertain Risks of Emerging Biotechnologies in the Netherlands. Risk Anal..

[10] Robaey Z., Spruit S., van de Poel I. (2017). The Food Warden: An Exploration of Issues in Distributing Responsibilities for Safe-by-Design Synthetic Biology Applications. Sci. Eng. Ethics.

[11] Amyotte P.R., Goraya A.U., Hendershot D.C., Khan F.I. (2007). Incorporation of Inherent Safety Principles in Process Safety Management. Process Saf. Prog..

[12] Robaey Z. (2018). Dealing with Risks of Biotechnology: Understanding the Potential of Safe-by-Design.

[13] Bollinger R.E., Clark D.G., Dowell R.M., Webank R.M., Hendershot D.C., Kletz T., Lutz W.K., Meszaros S.I., Park D.E., Wixom E.D. (1996). Inherently Safer Chemical Processes: A Life Cycle Approach.

[14] Edwards D.W. (2005). Are we too risk-averse for inherent safety? An examination of current status and barriers to adoption. Process Saf. Environ. Prot..

[15] van der Helm M.P., Bracco P., Busch H., Szymańska K., Jarzȩbski A.B., Hanefeld U. (2019). Hydroxynitrile lyases covalently immobilized in continuous flow microreactors. Catal. Sci. Technol..

[16] Coloma J., Guiavarc’h Y., Hagedoorn P.L., Hanefeld U. (2020). Probing batch and continuous flow reactions in organic solvents:Granulicella tundricolahydroxynitrile lyase (GtHNL). Catal. Sci. Technol..

[17] Coloma J., Lugtenburg T., Afendi M., Lazzarotto M., Bracco P., Hagedoorn P.-L., Gardossi L., Hanefeld U. (2020). Immobilization of Arabidopsis thaliana Hydroxynitrile Lyase (AtHNL) on EziG Opal. Catalysts.

[18] Keim W., Keim W. (2012). Catalysis in C1 chemistry.

[19] Buchner G.A., Stepputat K.J., Zimmermann A.W., Schomacker R. (2019). Specifying Technology Readiness Levels for the Chemical Industry. Ind. Eng. Chem. Res..

[20] Bracco P., Busch H., Von Langermann J., Hanefeld U. (2016). Enantioselective synthesis of cyanohydrins catalysed by hydroxynitrile lyases-a review. Org. Biomol. Chem..

[21] RIVM waterstofcyanide | | Risico’s van stoffen. https://rvszoeksysteem.rivm.nl/stof/detail/1325.

[22] RIVM Koolmonoxide|Risico’s van Stoffen. https://rvszoeksysteem.rivm.nl/stof/detail/859.

[23] Stankiewicz A.I., Moulijn J.A. (2000). Process intensification: Transforming Chemical Engineering. Chem. Eng. Prog..

[24] Löwe H., Ehrfeld W. (1999). State-of-the-art in microreaction technology: Concepts, manufacturing and applications. Electrochim. Acta.

[25] Sie S.T. (1996). Miniaturization of Hydroprocessing Catalyst Testing Systems: Theory and Practice. AIChE J..

[26] Turney R.D. Inherent Safety: What can be done to increase the use of the concept. Proceedings of the HJ Pasman: Loss Prevention and Safety Promotion in the Process Industries-10th International Symposium.

[27] Rusli R., Shariff A.M., Khan F.I. (2013). Evaluating hazard conflicts using inherently safer design concept. Saf. Sci..

[28] Schwarz-Plaschg C., Kallhoff A., Eisenberger I. (2017). Making Nanomaterials Safer by Design?. Nanoethics.

[29] Grundke C., Opatz T. (2019). Strecker reactions with hexacyanoferrates as non-toxic cyanide sources. Green Chem..

[30] Van Gorp A., Van De Poel I. (2001). Ethical considerations in engineering design processes. IEEE Technol. Soc. Mag..

[31] Swuste P., Groeneweg J., van Gulijk C., Zwaard W., Lemkowitz S., Oostendorp Y. (2020). The future of safety science. Saf. Sci..

[32] Rutherford W. (2003). Aviation safety: A model for health care?. Qual. Saf. Heal. Care.

[33] Singh N. (2009). On a wing and a prayer: Surgeons learning from the aviation industry. J. R. Soc. Med..

[34] Groeneweg J., Ter Mors E., Van Leeuwen E., Komen S. (2018). The Long and Winding Road to a Just Culture. Proceedings of the SPE International Conference and Exhibition on Health, Safety, Security, Environment, and Social Responsibility.

[35] Buchner G.A., Zimmermann A.W., Hohgrave A.E., Schomacker R. (2018). Techno-economic Assessment Framework for the Chemical Industry - Based on Technology Readiness Levels. Ind. Eng. Chem. Res..

[36] van der Berg J.P., Kleter G.A., Battaglia E., Bouwman L.M.S., Kok E.J. (2020). Application of the Safe-By-Design Concept in Crop Breeding Innovation. Int. J. Environ. Res. Public Health.

[37] Asin-Garcia E., Kallergi A., Landeweerd L., Martins dos Santos V.A.P. (2020). Genetic Safeguards for Safety-by-design: So Close Yet So Far. Trends Biotechnol..

[38] Casson Moreno V., Papasidero S., Scarponi G.E., Guglielmi D., Cozzani V. (2016). Analysis of accidents in biogas production and upgrading. Renew. Energy.

[39] Regårdh P. (2011). Safe, Secure and Ethical? Assessing and Regulating Risks Associated with Synthetic Biology. Ph.D. Thesis.

[40] Maurer S., Lucas K., Terrell S. (2006). From Understanding to Action: Community-Based Options for Improving Safety and Security in Synthetic Biology.

[41] Schmidt M., Dando M., Deplazes A. (2011). Dealing with the outer reaches of synthetic biology biosafety, biosecurity, IPR and ethical challenges of chemical synthetic biology. Chem. Synth. Biol..

[42] Knapland K.S., Knaplund K.S. (2011). Synthetic Cells, Synthetic Life, and Inheritance. Valpso. Univ. Law Rev..

[43] Domingo J.L., Nadal M. (2019). Human exposure to per- and polyfluoroalkyl substances (PFAS) through drinking water: A review of the recent scientific literature. Environ. Res..

